# *Lactobacillus crispatus*-Mediated Gut–Reproductive Tract Axis-Alleviated Microbial Dysbiosis and Oviductal Inflammation in a Laying Hen Model

**DOI:** 10.3390/microorganisms12081559

**Published:** 2024-07-30

**Authors:** Shinuo Li, Qingfeng Wang, Jinqiu Mi, Haotian Chen, Tianhao Yuan, Yue Wang, Lihong Zhao, Qiugang Ma, Shimeng Huang

**Affiliations:** 1 National Key Laboratory of Livestock and Poultry Nutrition and Feeding, College of Animal Science and Technology, China Agricultural University, Beijing 100193, China; seetshinuo857@outlook.com (S.L.); wangeangel@163.com (Q.W.); 15952235335@163.com (T.Y.); wangy07224@163.com (Y.W.); zhaolihongcau@cau.edu.cn (L.Z.); 2 Laboratory of Feedgrain Safety and Healthy Poultry Farming, Beijing Jingwa Agricultural Science and Technology Innovation Center, Beijing 101206, China

**Keywords:** *Lactobacillus crispatus*, hens, salpingitis, laying performance, microbiome, gut–reproductive tract axis

## Abstract

Oviductal inflammation (OI) significantly reduces the egg production and economic returns in poultry farming. While *Lactobacillus crispatus* (LAC) is effective against inflammation, its role in treating or preventing oviductal inflammation is understudied. In this study, we investigated the therapeutic mechanisms of LAC on oviductal inflammation, with a focus on reproductive tract health, microbiome, gene expression, and cytokine levels. This study involved 24 Jingfen No. 6 laying hens aged 60 weeks, divided into four groups: the CON, OI, OI + LAC, and OI + heat-killed *Lactobacillus crispatus* (HLAC) groups. And it included a 10-day adaptation, a 7-day period for the development of OI using inflammation-inducing drugs (the control received saline), followed by an 8-day treatment in which the CON and OI groups received 1 mL of MRS broth daily, and the OI + LAC and OI + HLAC groups were treated with live and heat-killed *Lactobacillus crispatus* (10^9^ CFUs/mL), respectively, with six hens in each group. This study showed that *Lactobacillus crispatus* supplementation significantly reduced the oviductal inflammation and atrophy in the hens, with the affected hens showing markedly lower egg production rates (*p* < 0.001) compared to the control and treated groups (OI + HLAC and OI + LAC). The daily intake of fresh (OI + LAC, *p* = 0.076) or heat-killed (OI + HLAC, *p* < 0.01) *Lactobacillus crispatus* notably enhanced the feed conversion efficiency. The OI group suffered significant ovarian damage and vascular rupture, more so than the CON group, while *Lactobacillus crispatus* supplementation mitigated this damage. The IL-1β, IL-6, and IL-8 levels were significantly elevated in the OI group compared to those in the OI + LAC group (*p* < 0.05), with a significant reduction in the TNF-α levels in the latter (*p* < 0.001). The supplementation improved the microbial composition in the cecum, isthmus, and shell gland, enriching the cecum with beneficial bacteria, such as *Ruminococcus_torques_group* and *Megamonas*. This approach fostered ovarian health and follicle differentiation and preserved the epithelial cell barrier function in the shell gland, reducing inflammatory damage in the genital tract. This dual efficacy underscores the role of the probiotic in diminishing oviductal inflammation, regardless of its state.

## 1. Introduction

Salpingitis in laying hens, defined as inflammation of the oviduct or infundibulum, often due to bacterial infection, is a common condition in the poultry industry. Its clinical manifestations typically include oviductal inflammation, broken eggshells, oophoritis, and peritonitis [1,2,3]. Given the conditions of large-scale intensive farming, laying hens frequently remain in suboptimal health states for extended periods, increasing their susceptibility to this prevalent disease [2]. Salpingitis primarily manifests as reduced egg production with poor eggshell quality, leading to thin, soft, or sandy shells, significantly threatening the health and economic viability of the laying hen industry and causing economic losses due to defects in the egg quality, making poultry eggs less available as a high-protein food source for humans [1,2,3,4]. The primary cause of salpingitis in laying hens can be attributed to pathogen infections in the oviduct, which are facilitated by the persistent mechanical stress from intensive egg laying and the subsequent dysbiosis and oviductal inflammation [2]. Characterized by its widespread outbreaks and high incidence rate, oviductal inflammation significantly diminishes laying performance, thereby causing substantial economic losses and raising food safety concerns [5]. For decades, numerous studies have reported a complex and mutually symbiotic relationship between laying hens and their carrying microbiota, especially their gut microbiota. Given that the gut microbiota of laying hens is positively associated with improvements in the production performance [6], egg quality [7], and host health [8], the alleviation of oviductal inflammation by studying the effects of microbiota supplementation seems feasible. Microorganisms play a crucial role in the pathogenesis of the disease, with infections likely occurring through the oviduct [5,9]. However, compared to the intestinal tract, there is scant research on the microbiota of the reproductive tract in relation to oviductal inflammation, indicating the need for further study in this area.

The billions of microbes within the vaginal microecosystem are also the critical determinants of vaginal health [10]. *Lactobacillus* species, which predominate in a healthy vaginal microbiome, produce various antimicrobial substances that are essential for maintaining female reproductive health [11]. These mechanisms include inhibiting pathogenic bacteria through competitive exclusion, antagonistic activities, and the production of lactic acid and bacteriocins [12,13]. In humans, the successful restoration of a vagina infected with bacterial vaginosis through the oral administration of *Lactobacillus crispatus* has been documented [14]. However, information regarding whether and how *Lactobacillus crispatus* improves the production performance and reproductive health of laying hens is limited. Similarly, the *Lactobacillus crispatus* effects on the serum biochemical changes during oviductal inflammation are not well understood. In this study, we hypothesized that *Lactobacillus crispatus* could serve as an effective alternative to antibiotics in preventing and treating oviductal inflammation. Among the *Lactobacillus* species, *Lactobacillus crispatus* is particularly significant for fertility and women’s health, providing protection against bacterial infections in the human vagina by resisting colonization through hydrogen peroxide production [15,16]. The lactic acid produced by *Lactobacillus crispatus* also contributes to a lower vaginal pH, thereby making the vagina an unfavorable environment for the growth of pathogenic microorganisms [17]. Furthermore, a lower pH means the inhibition of both dysbiosis and inflammation [18]. For example, Anton et al. highlight the anti-inflammatory effects induced by *Lactobacillus crispatus* in the cervicovaginal space under both normal and dysbiotic conditions. Based on this evidence, *Lactobacillus crispatus* was selected for supplementation in our experiment [19].

The main objective of this study was to verify the therapeutic effects of *Lactobacillus crispatus* on oviductal inflammation. We evaluated specific immune and inflammatory factors in oviductal inflammation-affected laying hens. This study involved 24 Jingfen No. 6 laying hens aged 60 weeks, with a particular focus on their gut microbiota, which allowed us to explore the potential relationship between *Lactobacillus crispatus* supplementation and the cecal, uterine, and isthmic microbiomes of laying hens.

## 2. Materials and Methods

### 2.1. Ethical Statement

This study was approved by the Animal Protection and Utilization Committee of China Agricultural University (AW92104202-1-5, Beijing, China), and it was conducted in accordance with the Guidelines for the Use of Experimental Animals of the Ministry of Science and Technology (Beijing, China).

### 2.2. Probiotic Strain Preparation

*Lactobacillus crispatus* (BNCC135057) was provided by the BeNa Culture Collection (Beijing, China), frozen at −80 °C, and activated in de Man, Rogosa, and Sharpe (MRS) broth (LuQiao Company, Beijing, China) at 37 °C for 12 h. After three passages, the strain’s concentration was adjusted to 1 × 10^9^ colony-forming units per milliliter (CFUs/mL). The culture was then centrifuged at 8000× *g* for 15 min at 4 °C, the supernatant was discarded, and the pellet was resuspended in one-tenth of the original volume with 0.85% normal saline to achieve a final concentration of 1 × 10^9^ CFUs/mL. The heat-killed strains were prepared by heating the suspensions at 95 °C for 30 min so that they were ready for use in further experiments.

### 2.3. Birds and Experiment Design

Twenty-four 60-week-old Jingfen No. 6 laying hens with uniform body weights and similar egg laying rates were selected and purchased from Huadu Yukou Poultry Co., Ltd., in Beijing, China, and were then assigned to four treatment groups: the CON, OI, OI + HLAC, and OI + LAC groups. Throughout the experiment, all of the groups were fed a basal diet, with the diet formulation as shown in Table 1. The experiment was conducted in three phases: first, a 10-day adaptation period; second, a 7-day period for the oviductal inflammation model creation, with the control group receiving sterile saline and the OI, OI + HLAC, and OI + LAC groups receiving daily inflammation-inducing drugs; and third, an 8-day treatment period during which the CON and OI groups were administered 1 mL of MRS broth daily using gavage, the OI + LAC group received fresh *Lactobacillus crispatus* (1 × 10^9^ CFUs mL^−1^), and the OI + HLAC group received heat-killed *Lactobacillus crispatus* (1 × 10^9^ CFUs mL^−1^), all administered in equivalent volumes using gavage once a day. For the construction of the laying hen oviductal inflammation model, the hens were inverted, 1 mL of 20% phenol mucilage was injected into their oviducts, and they were then kept inverted for 5 min to ensure effective distribution. Each treatment group consisted of six biological replicates, with one hen per replicate, and the hens were housed in individual stainless steel cages measuring 40 × 40 × 40 cm^3^ under controlled environmental conditions at 22 °C with a 16 h light/8 h dark cycle. The provided mash diets were based on corn and soybean meal, formulated according to the “Feeding Standard of Chicken, China (NY/T 33-2004)” [20], by the Chinese Ministry of Agriculture. Importantly, no anti-inflammatory drugs were administered during the probiotic treatment period in this study.

### 2.4. Sample Collection

At the end of the experiment (at 445 days of age), blood was drawn from each hen’s wing vein after 12 h of fasting and was then centrifuged at 4 °C (3000× *g* for 15 min), and the sera were stored at −80 °C for the serum parameter analysis. Following CO_2_ euthanasia, the abdomen was opened to extract the ovaries, infundibulum, shell gland, and cecum. The ovarian tissues were photographed for follicle counting. Samples measuring 0.5 cm from the infundibulum, shell gland, and cecum were fixed in 4% paraformaldehyde for the histological examination. These tissues, along with the cecal contents, were washed, flash-frozen in liquid nitrogen, and stored at −80 °C for further analysis.

### 2.5. Laying Performance

The laying rate and egg weight were recorded daily, and the average daily feed intake was recorded weekly per replicate. Additionally, the laying and feed efficiencies (feed conversion ratios (FCRs)) were calculated.

### 2.6. Egg Quality

During the final days of both the oviductal inflammation and treatment phases, ten eggs from each treatment group were collected to ensure a comprehensive assessment of the egg quality, which was evaluated using specialized instruments from ORKA Food Technology, Ltd., Ramat Hasharon, Israel (ESTG-1). These measurements included the shell strength, egg weight, albumen height, yolk color, and Haugh unit. The albumen height was measured at three or four locations and averaged for precision. The shell strength was gauged using an egg force tester (ESTG-1), and a multifunctional egg quality tester (EA-01, Orka Technology Ltd., Manchester, UK) was utilized to determine the Haugh unit and yolk color and to verify the egg weight. This structured approach ensured the consistency and reliability of the quality analysis.

### 2.7. Cecum and Oviduct Histopathology

During the necropsy, 0.5 cm segments of the cecum, uterus, and isthmus tissues from each chicken were longitudinally sectioned. These tissues were washed with a 0.9% NaCl solution and then immediately fixed in 4% (*v*/*v*) paraformaldehyde in PBS until further processing. Post-fixation, the cecum, uterus, and isthmus samples underwent graded ethanol processing and were embedded in paraffin, sectioned into 5 µm thick slides with a microtome (Leica Microsystems K.K., Tokyo, Japan), and stained with Hematoxylin and Eosin (H&E, Baton Rouge, LA, USA). The histological parameters were analyzed using computer-assisted microscopy (Nikon ECLIPSE E200, Nikon, Tokyo, Japan).

### 2.8. Observation of Follicles

At the end of the experiment, six hens, with six replicates per treatment group, were selected for slaughter. Immediately after the slaughtering, the oviduct, ovary, and follicular hierarchies were excised and weighed using an analytical balance. The pre-hierarchical follicles were classified into four groups based on their morphologies and diameters: small white follicles (SWFs) (<5 mm); large white follicles (LWFs) (from 5 to 8 mm); small yellow follicles (SYFs) (from 8 to 10 mm); and large yellow follicles (LYFs) (from 10 to 12 mm). 

### 2.9. Serum and Uterus Inflammatory Index Detection

At the end of the experiment, 6 hens from each treatment group, totaling 24 birds across the 4 groups, were chosen and euthanized via cervical dislocation to ensure humane handling and compliance with ethical standards. Blood samples from the jugular vein were collected into 10 mL tubes, left to stand for 2–3 h, and then centrifuged at 3000 r/min for 15 min to collect the serum, which was stored at −80 °C. After the blood collection, a 5–8 cm segment of the uterus was excised, quickly frozen in liquid nitrogen, and also stored at −80 °C. For the analysis, the uterus was homogenized in saline, and the supernatant was centrifuged at 3000 r/min for 10 min. The serum and uterine tumor necrosis factor-α (TNF-α), interleukin-1β (IL-1β), interleukin-6 (IL-6), and interleukin-8 (IL-8) levels were measured using ELISA kits (Nanjing Jiancheng Bioengineering Institute, Nanjing, China), following the kit’s instructions.

### 2.10. RNA Extraction and Real-Time Quantitative PCR

Total RNA samples were obtained from the uterus using the FastPure cell/Tissue Total RNA Isolation Kit V2 (Vazyme Biotech Co., Ltd., Nanjing, China) and were then reverse-transcribed into cDNA using the HiScript II Q RT SuperMix for qPCR Kit (R223-01, Vazyme Biotech Co., Ltd.) with the gDNA wiper Kit (Takara, Beijing, China), in accordance with the manufacturer’s instructions. The mRNA expression levels of the barrier function and inflammation genes in the uterus were evaluated. The mRNA expression was assessed using a commercially available kit (Takara). The qPCR protocol comprised denaturation at 95  °C for 1 min, followed by 40 cycles of 15 s at 95  °C and 15 s at 60  °C. The housekeeping gene glyceraldehyde-3-phosphate dehydrogenase (GAPDH) was used as the internal reference gene. The primer sequences are shown in Table S1.

### 2.11. DNA Extractions and 16S rRNA Gene Sequencing of Cecal, Uterine, and Isthmic Microbiota

Total DNA from the cecal, uterine, and isthmic microbiota of each bird was extracted using the E.Z.N.A. Soil DNA Kit (Omega Bio-tek, Norcross, GA, USA), following the manufacturer’s instructions. A PCR was used to amplify the V4 region (515F-806R) of the bacterial 16S rRNA gene. The PCR reactions were conducted in a 30 µL mix with 15 µL of Phusion High-Fidelity PCR Master Mix (New England Biolabs, Ipswich, MA, USA), 0.2 µM of each primer, and 10 ng of the template DNA. The PCR protocol included an initial denaturation at 98 °C for 1 min, followed by 30 cycles of 98 °C for 10 s, 50 °C for 30 s, and 72 °C for 30 s, and a final extension at 72 °C for 5 min. The PCR products, mixed with 1 × loading buffer (with SYBR green), were electrophoresed on a 2% agarose gel. The products were normalized by density, purified using the GeneJET Gel Extraction Kit (Thermo Scientific, Waltham, MA, USA), and used to prepare sequencing libraries with the Ion Plus Fragment Library Kit 48 rxns (Thermo Scientific), adhering to the manufacturer’s guidelines. The library quality was evaluated on a Qubit@ 2.0 Fluorometer (Thermo Scientific). The sequencing was performed on the MiSeq platform using the Miseq Reagent Kit v3, provided by Shanghai Personal Biotechnology Co., Ltd. In Shanghai, China. 

### 2.12. Analysis of Cecal, Uterine, and Isthmic Microbiota

The microbial community profiling involved the demultiplexing and quality filtering of the raw paired-end sequences with QIIME (version 2.0). Sequences with overlaps of >10 base pairs were merged based on their sequence overlap. Amplicon sequence variants (ASVs) were clustered at 97% sequence similarity using UPARSE (version 7.1), with the chimeric sequences identified and removed using UCHIME (version 2.0), producing effective tags. The Mann–Whitney U test was used to evaluate the statistical significance of the alpha diversity metrics. A principal coordinate analysis (PCoA) was used to analyze the bacterial-community structures across the samples, with significant differences between the groups tested via an analysis of similarity (ANOSIM) and non-parametric multivariate ANOVA (ADONIS) using the Vegan package for the Bray–Curtis matrix, PCoA, and ADONIS calculations. R software (version 4.2.2) was used to generate Venn diagrams and bacterial relative abundance charts. Alpha diversity indices, including the Ace, Chao, Shannon, Simpson, and Sobs indices, were analyzed using Mothur (version 1.30), with the significance assessed using the Mann–Whitney U test. Two-sided Student’s *t*-tests were used to determine the bacterial community composition differences between the groups. The linear discriminant analysis (LDA) effect size (LEfSe) method was used to identify differentially abundant taxa, setting an alpha of 0.05 for the Kruskal–Wallis test among the classes and an LDA score threshold (log10 LDA) of 2.5 to estimate each feature’s effect size. Significant differences among the groups were indicated by *p* < 0.05.

### 2.13. Statistical Analysis

Data are expressed as means ± SEMs. Statistical analyses were carried out using a one-way ANOVA followed by Tukey’s multiple-comparison test to identify the significant differences between the groups. Data visualization and statistical analyses were performed using GraphPad Prism 8.0, with statistical significance categorized as *p* < 0.05, *p* < 0.01, and *p* < 0.001.

## 3. Results

### 3.1. Effect of Lactobacillus crispatus on the Laying Performance of Laying Hens during Oviductal Inflammation

**Figure 1:**
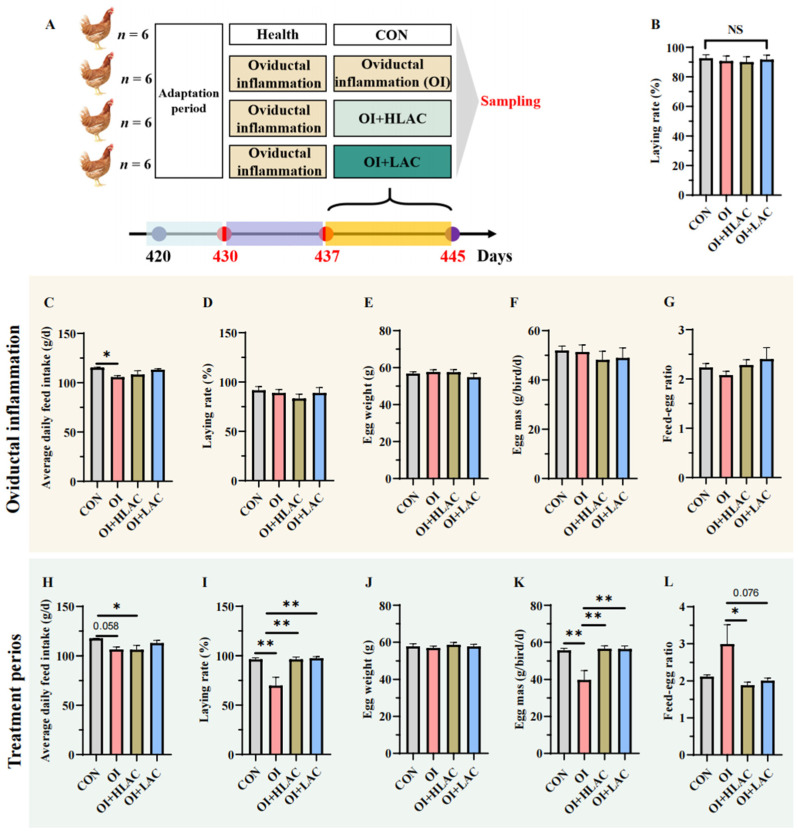
Effects of *Lactobacillus crispatus* on the laying performance of laying hens. (**A**) Experimental design. The experiment was conducted in three phases: first, a 10-day adaptation period; second, a 7-day period for the oviductal inflammation model creation, with the control (CON) group receiving sterile saline and the oviductal inflammation (OI), OI + heat-killed *Lactobacillus crispatus* (HLAC), and OI + *Lactobacillus crispatus* (LAC) groups receiving daily inflammation-inducing drugs; and third, an 8-day treatment period during which the CON and OI groups were administered 1 mL of MRS broth daily using gavage, the OI + LAC group received fresh *Lactobacillus crispatus* (1 × 10^9^ CFUs mL^−1^), and the OI + HLAC group received heat-killed *Lactobacillus crispatus* (1 × 10^9^ CFUs mL^−1^), all administered in equivalent volumes using gavage once a day. (**B**) Average laying rate of laying hens during the adaptation period. (**C**–**G**) Average daily feed intake (**C**), laying rate (**D**), egg weight (**E**), egg mass (**F**), and feed–egg ratio (**G**) of laying hens during oviductal inflammation period. (**H**–**L**) Average daily feed intake (**H**), laying rate (**I**), egg weight (**J**), egg mass (**K**), and feed–egg ratio (**L**) during the treatment phase. Data are expressed as means ± SEMs (*n*  =  6). * *p* < 0.05, ** *p* < 0.01.

The experimental design is shown in Figure 1A. The average daily feed intake, laying rate, egg weight, egg mass (g/hen/d), and feed–egg ratio of the layers were calculated (Figure 1). Before inflammatory treatment to induce oviductal inflammation, there was no significant difference in the laying rates between the four groups (Figure 1B). During the period of induced oviductal inflammation in the laying hens, no significant differences were observed in the laying rate, egg weight, egg mass, or feed–egg ratio (Figure 1D–G). The average daily feed intake in the OI group was significantly lower than that in the CON group (*p* < 0.05) (Figure 1C). During the treatment period, the average feed intake in the OI group still tended to be lower than that in the CON group (*p* = 0.058) (Figure 1H). The laying rate in the OI group was significantly lower than those in the CON, OI + HLAC, and OI + LAC groups (*p* < 0.001) (Figure 1I). There was no significant difference in the egg weights between the four groups (Figure 1J). Therefore, the feed–egg ratio in the OI + HLAC (*p* < 0.01) and OI + LAC (*p* = 0.076) groups was lower than that in the OI group (Figure 1L).

### 3.2. Effects of Lactobacillus crispatus on Laying Hen Egg Quality during Oviductal Inflammation

**Figure 2:**
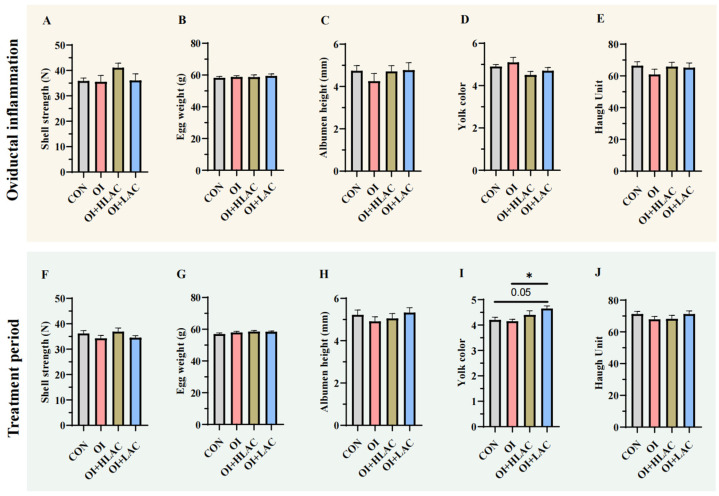
Effects of *Lactobacillus crispatus* on laying hen egg quality. (**A**–**E**) Measurements of shell strength (**A**), egg weight (**B**), albumen height (**C**), yolk color (**D**), and Haugh unit (**E**) for laying hens during oviductal inflammation period. (**F**–**J**) Observations of shell strength (**F**), egg weight (**G**), albumen height (**H**), yolk color (**I**), and Haugh unit (**J**) for laying hens during treatment period. Data are expressed as means ± SEMs (*n*  =  10). * *p* < 0.05.

As shown in Figure 2, there were no significant differences among the groups in terms of the shell strength, egg weight, albumen height, yolk color, or Haugh unit during the oviductal inflammation period (*p* > 0.05) (Figure 2A–E). During the treatment period, the yolk color of the OI + LAC group was higher (*p* < 0.01) than that of the OI group during the treatment period (Figure 2I). In addition, the yolk color of the LAC group was higher than that of the control (*p* = 0.05) (Figure 2I). However, no significant differences were observed in terms of the shell strength, egg weight, albumen height, and Haugh unit (*p* > 0.05) (Figure 2F–H,J).

### 3.3. Effect of Lactobacillus crispatus on Laying Hen Ovarian Health during Oviductal Inflammation

**Figure 3:**
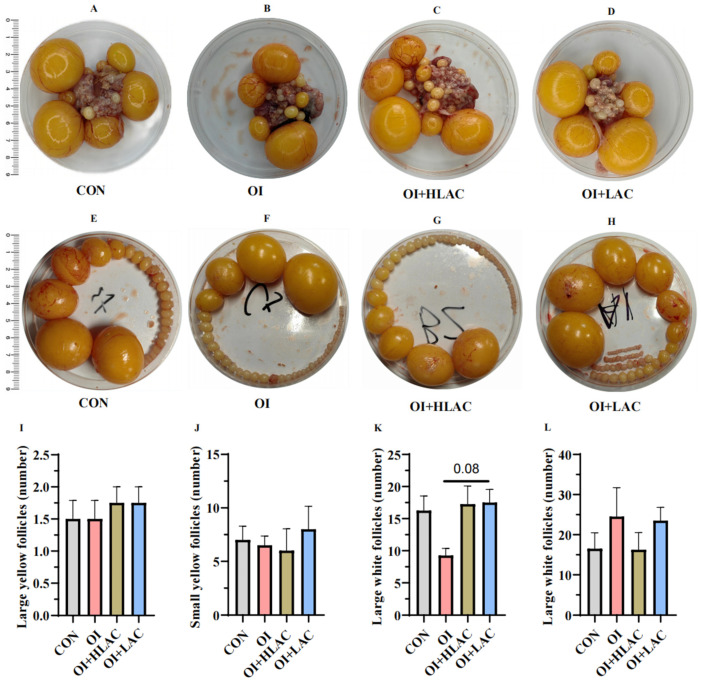
Effects of *Lactobacillus crispatus* on laying hen ovarian health during oviductal inflammation. (**A**–**D**) Ovarian states of laying hens across various treatment groups. (**E**–**H**) Follicle counts of varying grades in laying hens subjected to different treatments, including LYFs (large yellow follicles) (**I**), SYFs (small yellow follicles) (**J**), LWFs (large white follicles) (**K**), and SWFs (small white follicles) (**L**). Data are shown as means ± SEMs (*n*  =  6).

As shown in Figure 3, although the follicles extracted from the oviducts of the laying hens across all the groups appeared to be well formed and were surrounded by smooth blood vessels, notable differences were observed in the condition of the ovarian tissue. Specifically, the OI group experienced significant ovarian atrophy and vascular rupture, indicating a higher degree of damage compared to the control group. In contrast, the OI + HLAC, and OI + LAC groups exhibited varying degrees of improvement, with reduced damage to the ovarian tissue. We then counted the total number of follicles in each group (Figure 3I–K). No remarkable changes were observed among the four groups (*p* > 0.05). However, the number of large white follicles in the OI + LAC group tended to increase compared with those of the OI group, which had the smallest number (*p* = 0.08) (Figure 3K).

### 3.4. Effect of Lactobacillus crispatus on Inflammatory Cytokines in the Serum and Uterus of Laying Hens during Oviductal Inflammation

**Figure 4:**
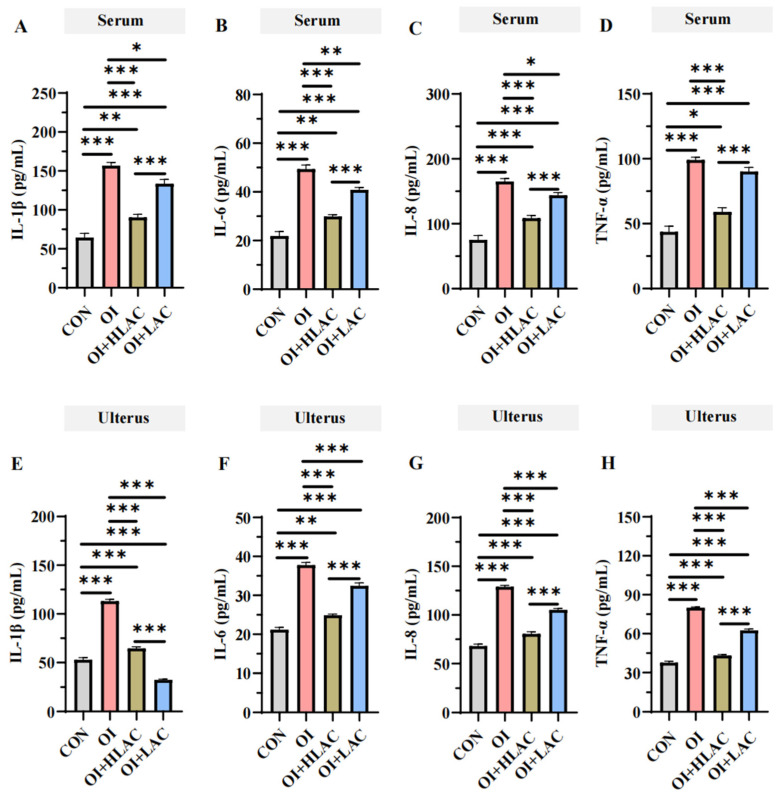
Effects of *Lactobacillus crispatus* on inflammatory cytokines in laying hen serum and uterus during oviductal inflammation. (**A**–**D**) IL-1β (**A**), IL-6 (**B**), IL-8 (**C**), and TNF-α (**D**) levels in laying hens during oviductal inflammation period. (**E**–**H**) IL-1β (**E**), IL-6 (**F**), IL-8 (**G**), and TNF-α (**H**) levels in laying hens during treatment period. IL-1β, interleukin-1β; IL-6, interleukin-6; IL-8, interleukin-8; TNF-α, tumor necrosis factor-α. Data are expressed as means ± SEMs (*n*  =  6). * *p* < 0.05, ** *p* < 0.01, *** *p* < 0.001.

As shown in Figure 4, the IL-1β, IL-6, IL-8, and TNF-α concentrations were significantly elevated in the serum and uterine tissues of the OI group compared to those in the CON (control), OI + LAC, and OI + HLAC groups (*p* < 0.001). Specifically, the IL-1β, IL-6, and IL-8 levels in the serum and uterine tissues were notably higher in the OI group than those in the OI + LAC and OI + HLAC groups (*p* < 0.05). Additionally, the TNF-α levels in the uterine tissue were significantly greater in the OI group compared to those in the OI + LAC and OI + HLAC groups (*p* < 0.001). When compared to the CON group, the OI group exhibited significantly higher = IL-1β, IL-6, IL-8, and TNF-α levels in the uterine tissue (*p* < 0.001). Crucially, these levels significantly decreased in the OI + LAC group when compared to the OI group (*p* < 0.001). Furthermore, the IL-1β, IL-6, IL-8, and TNF-α levels in the uterine tissue were significantly reduced in the OI + LAC group compared to those in the OI + HLAC group (*p* < 0.001).

### 3.5. Effect of Lactobacillus crispatus on the Histological Morphology of Cecum, Isthmus, and Shell Gland Tissues and Inflammation-Related Gene Expression in the Shell Glands of Laying Hen Serum and Uterus during Oviductal Inflammation

**Figure 5:**
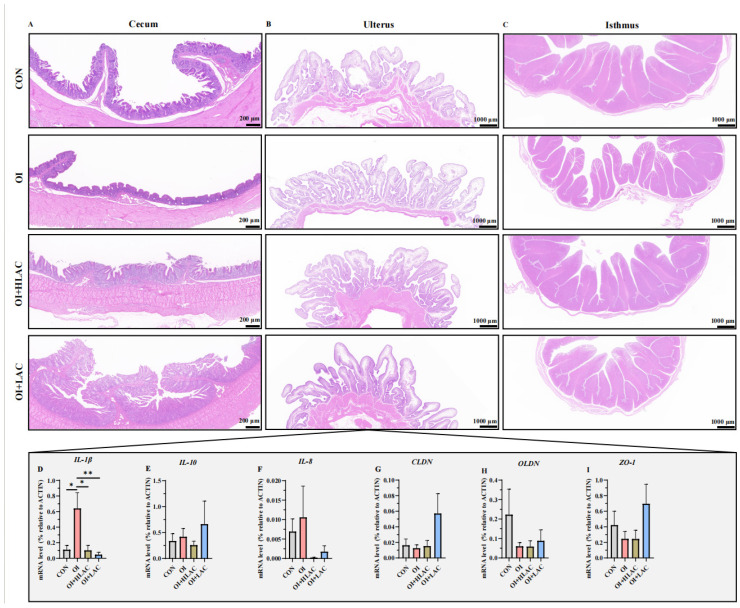
Effects of *Lactobacillus crispatus* on histological morphology of cecum (**A**), isthmus (**B**), and shell gland (**C**) tissues and inflammation-related gene expression (**D**–**I**) in the shell glands of laying hens during oviductal inflammation. IL-1β, interleukin-1β; IL-10, interleukin-10; IL-8, interleukin-8; CLDN, claudin; OLDN, Occludin; ZO-1, Zonula occludens-1. Data are expressed as means ± SEMs (*n*  =  3). * *p* < 0.05, ** *p* < 0.01.

Histological sections of cecum, uterus, and isthmus tissues were obtained (Figure 5). The brush border of the cecum was intact in the CON group, while it was thinner in the OI group (Figure 5A). The OI group had the smallest cecal gland number and size. The muscularis in the cecum in the OI + HLAC and OI + LAC groups was thicker, and the circular muscle was much more developed. The structure of the uterus tissue was complete and clear without inflammatory cell infiltration, vacillation, or cell death in the CON, OI + HLAC, and OI + LAC groups (Figure 5B). Among these three groups, the OI + HLAC group had the least thick muscular layer. The OI group had a small amount of inflammatory cell infiltration, a thinner basal layer of the endometrium, and a smaller portion of the myometrium with unclear stratification. In addition, some of the tubal epithelial cells shed on the endometrium in the OI group. The structure of the isthmus tissue was whole in the CON and OI + LAC groups, while the basal layer was incomplete in the OI and OI + HLAC groups (Figure 5C). Obvious inflammatory cell infiltration and scattered tissue were shown in the OI group, while those in the OI + HLAC and OI + LAC groups were decreased.

We subsequently evaluated the variations in the secretion levels of the barrier-related genes and inflammatory cytokines in the laying hens, as illustrated in Figure 5D–I. Compared to the OI group, significant decreases in the IL-1β mRNA expression were observed in the CON, OI + HLAC (*p* < 0.05), and OI + LAC (*p* < 0.01) groups. However, the IL-10, IL-8, CLDN, OLDN, and ZO-1 levels showed no significant differences (*p* > 0.05).

### 3.6. Effects of Lactobacillus crispatus on Cecal, Uterine, and Isthmic Microbiota in Laying Hens during Oviductal Inflammation

**Figure 6:**
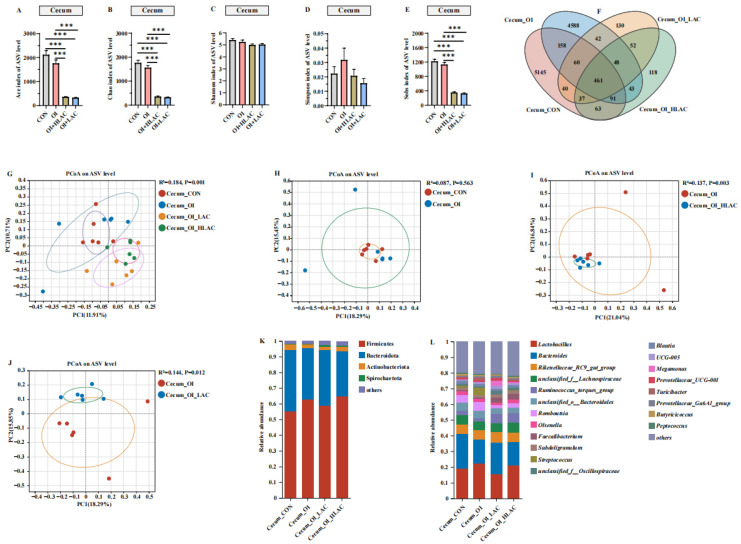
Effects of *Lactobacillus crispatus* on cecum microbiota in laying hens during oviductal inflammation. (**A**–**E**) Measures of five alpha diversity metrics (Ace, Chao, Shannon, Simpson, and Sobs indices) in laying hen ceca. (**F**) Venn diagram illustrating Amplicon Sequence Variant (ASV) overlap. (**G**–**J**) Principal coordinate analysis (PCoA) plots based on weighted UniFrac distances depicting microbiota similarity across different groups. (**K**) Chart detailing average distribution of major phyla within cecal microbiota. (**L**) Chart presenting average proportion of predominant genera in cecal microbiota. Data are expressed as means ± SEMs (*n*  =  6). *** *p* < 0.001.

**Figure 7:**
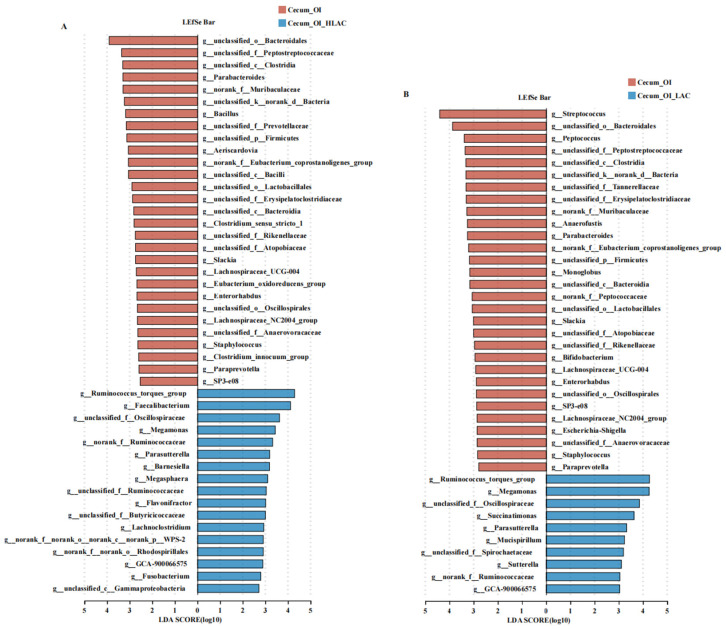
Effects of *Lactobacillus crispatus* on differential cecal microbes in laying hens during oviductal inflammation. (**A**) The linear discriminant analysis (LDA) effect size (LEfSe) analysis of the cecum microbiota between Cecum_OI (red) and Cecum_OI_HLAC (blue) groups at the genus level. (**B**) The LEfSe analysis of the cecum microbiota between Cecum_OI (red) and Cecum_OI_LAC (blue) groups at the genus level.

As shown in Figure 6, the alpha diversity was assessed using the Ace and Chao indices and showed that the CON group had the highest species richness and the most abundant read count, with over 2000 species identified, while the OI + HLAC and OI + LAC groups had the least richness (Figure 6). The Ace, Sobs, and Chao indices of the cecum in the OI group were significantly higher than those in the OI + HLAC and OI + LAC groups (*p* < 0.001). However, no remarkable changes were noted in the observed Shannon and Simpson indices among these four groups (*p* > 0.05). The Venn diagram presented in Figure 6F illustrates the distribution of the bacterial community ASVs. The four groups shared the same intestinal bacteria community, including 461 common ASVs. The CON group shared the same intestinal bacteria community as that of the OI group, including 770 ASVs. The numbers of unique ASVs in the CON, OI, OI + HLAC, and OI + LAC groups were 5145, 4588, 118, and 130, respectively. To further estimate the changes in the microbiota with the *Lactobacillus crispatus* addition, a PCoA analysis was used to visualize the beta diversity of the cecal microbiota in the laying hens (Figure 6G). There were significant differences in the beta diversity between the OI and OI + LAC groups, and the first two main axes explained 18.29% and 15.85% of the sample variance data. Next, the cecum microbiota composition was analyzed at the phylum and genus levels. The most abundant and prevalent phyla observed across all four groups included *Firmicutes*, followed by *Bacteroidetes* and smaller portions of *Actinobacteria* and *Spirochaetota* (Figure 6K). At the genus level, *Lactobacillus, Bacteroides,* and *Rikenellaceae_RC9_gut_group* were found to be the most abundant groups (Figure 6L). We then used LEfSe analysis to identify the bacteria at the species level that accounted for the greatest differences in the abundance in the cecum between the OI and OI + HLAC groups (Figure 7A) and between the OI and OI + LAC groups (Figure 7B). The differences in the taxa between the two groups were detected using linear discriminant analysis (LDA). Seventeen species, including *Ruminococus_torques_group*, *Faecalibacterium*, *Megamonas*, *Parasutterella*, *Barnesiella*, and *Megasphaera*, were significantly enriched in the OI + HLAC group, while *Parabacteroides*, *Bacillus*, *Aeriscardovia*, *Clostridium_sensu_stricto_1*, and *Slackia* were more prevalent in the OI group. Ten species, namely *Ruminococcus_torques_group*, *Megamonas*, *Succinatimonas*, *Parasutterella*, *Mucispirillum*, and *Sutterella*, were significantly enriched in the OI + LAC group, while *Streptococcus*, *Peptococcus*, *Anaerofustis*, *Parabacteroides*, *Monoglobus*, *Slackia*, and *Enterorhabdus* were more enriched in the OI group.

**Figure 8:**
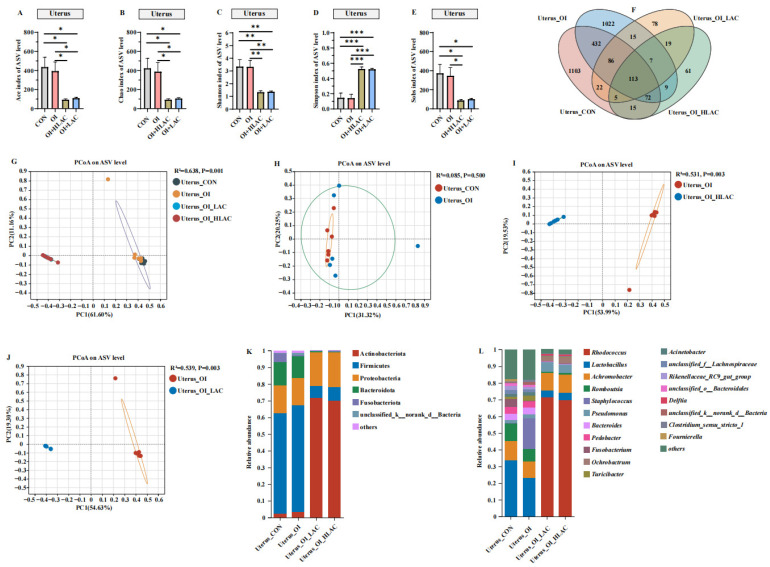
Effects of *Lactobacillus crispatus* on uterine microbiota of laying hens during oviductal inflammation. (**A**–**E**) Measures of five alpha diversity metrics (Ace, Chao, Shannon, Simpson, and Sobs indices) in laying hen ceca. (**F**) Venn diagram illustrating Amplicon Sequence Variant (ASV) overlap. (**G**–**J**) Principal coordinate analysis (PCoA) plots based on weighted UniFrac distances depicting microbiota similarity across different groups. (**K**) Chart detailing average distribution of major phyla within cecal microbiota. (**L**) Chart presenting average proportion of predominant genera in cecal microbiota. Data are expressed as means ± SEMs (*n*  =  6). * *p* < 0.05, ** *p* < 0.01, *** *p* < 0.001.

**Figure 9:**
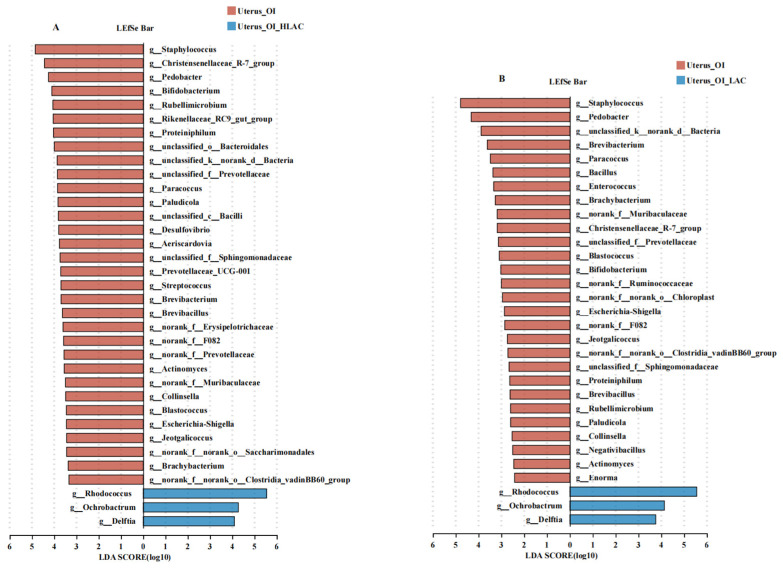
Effects of *Lactobacillus crispatus* on differential uterine microbes of laying hens during oviductal inflammation. (**A**) The LEfSe analysis of the uterine microbiota between Uterus_OI (red) and Uterus_OI_HLAC (blue) groups at the genus level. (**B**) The LEfSe analysis of the uterine microbiota between Uterus_OI (red) and Uterus_OI_LAC (blue) groups at the genus level.

As shown in Figure 8, no significant differences were observed in the alpha diversity indices between the OI + HLAC group and OI + LAC group, nor between the CON group and OI group (*p* > 0.05). The observed Ace, Chao, Shannon, and Sobs indices were significantly higher in the OI group than those in the OI + HLAC group (*p* < 0.01). The observed Ace, Chao, and Shannon indices were significantly higher in the OI group than those in the OI + LAC group (*p* < 0.01). The Simpson index showed that both the CON group and OI groups were the most diverse groups, while the OI + HLAC and OI + LAC groups had the least diversity. The Venn diagram shows that all four groups shared 113 common ASVs (Figure 8F). The numbers of unique ASVs in the CON, OI, OI + HLAC, and OI + LAC groups were 1103, 1022, 61, and 78, respectively (Figure 8F). Subsequently, a principal coordinate analysis (PCoA) was used to further analyze the beta-diversity differences among the groups. There was a clear separation between the OI and treatment groups, except for the CON group, for which the ADONIS *p*-value is 0.001 (Figure 8G), demonstrating significant LAC and HLAC influences on the uterine microbiota. At the phylum level (Figure 8K), the results show that Firmicutes was the most abundant bacteria in the CON and OI groups, whereas Actinobacteriota was the most abundant bacteria in the OI + HLAC and OI + LAC groups. At the genus level (Figure 8L), *Lactobacillus*, *Pseudomonas*, *Achromobacter*, and *Romboustia* were found to be the most abundant genera in the CON and OI groups, while *Rhodococus* was the most abundant genus in the OI + HLAC and OI + LAC groups, accounting for >70% of the total uterine microbial community. Next, LEfSe analysis was also conducted to identify the differentially abundant bacterial ASVs in the uterine microbiota between the OI and OI + HLAC groups (Figure 9A) and between the OI and OI + LAC groups (Figure 9B). Compared with the OI + HLAC group, *Staphylococcus*, *Christensenellaceae_R-7_group*, *Pedobacter*, *Bifidobacterium*, *Rikenellaceae_RC9_gut_group*, *Proteiniphilum*, *Paracoccus*, *Paludicola*, *Prevotellaceae_UC001*, *Streptococcus*, *Brevibacterium*, and *Brevibacillus* were more enriched in the OI group. Compared with the OI + LAC group, *Staphylococcus*, *Pedobacter*, *Brevibacterium*, *Paracoccus*, *Bacillus*, *Enterococcus*, *Brachybacterium*, *Christensenellaceae_R-7_group*, *Blastococcus*, *Escherichia-Shigella*, *Proteiniphilum*, and *Brevibacillus* were more prevalent in the OI group. The three species *Rhodococus*, *Ochrobactrum*, and *Delftia* were significantly enriched in both the OI + HLAC and OI + LAC groups compared with the OI group.

**Figure 10:**
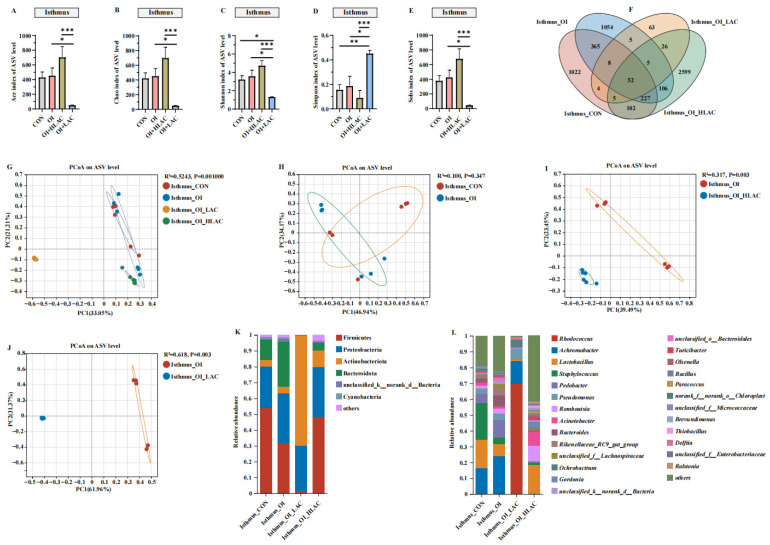
Effects of *Lactobacillus crispatus* on isthmus microbiota of laying hens during oviductal inflammation. (**A**–**E**) Measures of five alpha diversity metrics (Ace, Chao, Shannon, Simpson, and Sobs indices) in laying hen ceca. (**F**) Venn diagram illustrating Amplicon Sequence Variant (ASV) overlap. (**G**–**J**) Principal coordinate analysis (PCoA) plots based on weighted UniFrac distances depicting microbiota similarity across different groups. (**K**) Chart detailing average distribution of major phyla within cecal microbiota. (**L**) Chart presenting average proportions of predominant genera in cecal microbiota. Data are expressed as means ± SEMs (*n*  =  6). * *p* < 0.05, ** *p* < 0.01, *** *p* < 0.001.

**Figure 11:**
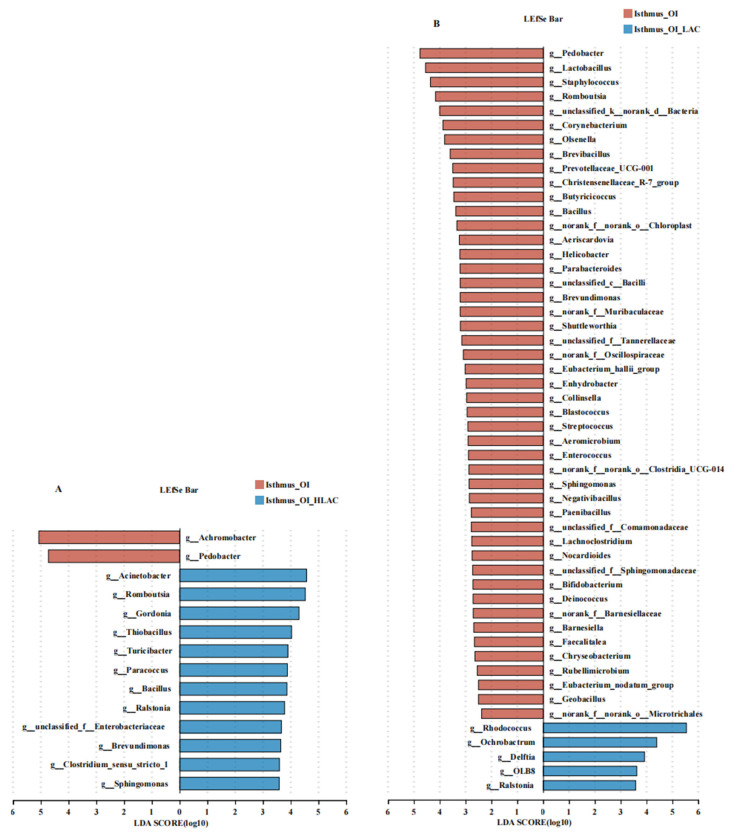
Effects of *Lactobacillus crispatus* on differential isthmus microbes of laying hens during oviductal inflammation. (**A**) The LEfSe analysis of the uterine microbiota between Isthmus_OI (red) and Isthmus_OI_HLAC (blue) groups at the genus level. (**B**) The LEfSe analysis of the uterine microbiota between Isthmus_OI (red) and Isthmus_OI_LAC (blue) groups at the genus level.

As shown in Figure 10, all of the alpha diversity indices for the OI + HLAC and OI + LAC groups showed significant differences (*p* < 0.001). Additionally, the observed Simpson index was the highest, and the Ace, Chao, Shannon, and Sobs indices were the lowest in the OI + LAC group. The shared and unique ASV proportions in the four groups are intuitively indicated in the Venn diagram in Figure 10F. There were 52 shared ASVs contained in the CON, OI, OI + HLAC, and OI + LAC groups; the numbers of unique ASVs were 1024, 1054, 2599, and 63, respectively. At the phylum level (Figure 10K), the results show that *Firmicutes* was the most abundant bacteria in the CON, OI, and OI + HLAC groups, whereas *Actinobacteriota* was the most abundant bacteria in the OI + LAC group. At the genus level (Figure 10L), *Rhodococcus* was the most abundant genus in the OI + LAC group. PCoA based on weighted UniFrac distances was utilized to measure the b-diversity. The PCoA results show the separation of the isthmic microbial communities between the CON and OI groups, and between the OI and OI + LAC groups, while the isthmus microbiota were aggregated in the CON and OI groups. According to the LEfSe analysis, in the OI group (Figure 11A), *Achromobacter* and *Pedobacter* were the characteristic bacteria, whereas in the OI + HLAC group, *Acinetobacter*, *Romboutsia*, *Gordonia*, *Thiobacillus*, *Turicibacter*, *Paracocus*, *Bacillus*, *Ralstonia*, *Brevundimonas*, *Clostridium_sensu_stricto_1*, and *Sphingomonas* were more characteristic. *Pedobacter*, *Lactobacillus*, *Staphylococus*, *Romboutsia*, *Corynebacterium*, and *Olsenella* were the characteristic bacteria in the OI group (Figure 11B), whereas in the OI + LAC group, *Rhodococcus*, *Ochrobactrum*, *Delftia*, *OLB8*, and *Ralstonia* were more distinctive.

## 4. Discussion

Improving or maintaining the egg quality and laying consistency to extend laying hen production cycles can bring substantial economic and environmental benefits, fostering a more sustainable egg industry. However, salpingitis, linked to the production of eggs with abnormal shapes and broken and soft shells, significantly affects the laying performance and egg quality [21]. This disease poses a widespread threat to laying hen farms, with consequences that include low egg production due to an uneven feed supply, eggshell rupture, oviduct damage, stress reactions, and diseases such as avian influenza, Newcastle disease, infectious bronchitis, avian leukemia, and egg drop syndrome. Additionally, poor hygiene in poultry houses and the infection of the cloaca with Gram-negative bacteria, especially *Salmonella* and *Escherichia coli*, are key contributors to oviduct infections. These pathogens secrete secondary metabolites that damage the intestinal or oviduct epithelial cells, thereby inducing inflammatory damage. Based on previous research [22,23] and preliminary experiments conducted by the research group, 1 mL of 20% phenol mucilage was injected into the oviducts of hens, and the hens were then kept inverted for 5 min to ensure effective distribution. This method aimed to simulate oviductal inflammation and damage to a certain extent. This study investigated the effects of severe chemical burns on the inflammatory state, oviductal and cecal histology, cytokine profiles, and microbiota of various regions of hens. Before 2018, China primarily controlled salpingitis in laying hens by adding antibiotics to their feed, which mitigated the disease but raised concerns about antibiotic residues and drug-resistant bacteria which pose risks to human health. Following the 2018 ban on antibiotics in poultry feed, maintaining reproductive-tract health has become crucial for the economic success of egg production. Given these challenges, finding alternatives to antibiotics to improve the health of laying hens is urgently needed.

Probiotics have had positive effects on improving animal health and performance [24]. Among these, *Lactobacillus* is a widely used probiotic, serving as an alternative to antibiotics for nutritional regulation against inflammation. There is a pressing need in the poultry industry to prevent salpingitis through dietary management, with *Lactobacillus* playing a key role in improving the laying rates [6]. Lv et al. reported that *Lactobacillus crispatus* supplementation in feed significantly decreased the FCR and increased the albumen height and Haugh unit, which aligns with our observations. These results suggest that *Lactobacillus crispatus* has some beneficial effects on improving the laying hen production performance and egg quality [25]. Furthermore, the introduction of *Lactobacillus* into diets has been shown to reduce the feed–egg ratio, indicating enhanced economic efficiency and health benefits. The egg quality, a critical aspect of production, was also assessed. Wan et al. reported no significant impact on the external egg quality from *Limosilactobacillus oris* supplementation, except in terms of the yolk weight [6]. In the present study, we observed similar outcomes. The yolk color, which is vital to consumer preferences due to its sensory appeal, was significantly enhanced by the *Lactobacillus crispatus* supplementation in our study, potentially due to its impact on the lipid metabolism and antioxidant properties in combating oxidative stress induced by salpingitis [26,27]. Thus, follicles are the basic functional units of ovaries, and their growth and development are important determinants of poultry egg production and, therefore, the fertility of hens [28]. Considering the pivotal role of ovaries and follicles in reproduction and egg production, our focus was on the follicle quantity and quality. We noted an increase in large white follicles and a trend towards more small yellow follicles in the Lactobacillus-treated groups, indicative of improved laying rates and oviduct health, which is a finding that is supported by Lu et al. [29]. This emphasizes the potential of *Lactobacillus crispatus* in enhancing the reproductive health and egg quality of poultry.

*Lactobacillus* modulates inflammatory responses by enhancing the mucosal barrier function and the phagocytosis of phagocytes, stimulating immune cells to produce anti-inflammatory cytokines and antibodies and inhibiting pro-inflammatory cytokine secretion, and it is effective against *E. coli* infections via the regulation of the intestinal immune function and for treating related infections. Salpingitis is characterized by oviduct distension, inflammation, and the accumulation of caseous exudate, leading to morphological and functional changes in the cecum, uterus, and isthmus, such as a compromised intestinal barrier, inflammatory cell infiltration, and cellular necrosis [1]. H&E staining has revealed that *Lactobacillus crispatus* supplementation mitigates these injuries and preserves the intestinal and reproductive tract integrity [30]. Pro-inflammatory cytokines disrupt the mucosal barrier function, with inflammatory factors at the mRNA and protein levels reflecting the ovarian function and health. In this study, we showed that oviductal inflammation induced inflammation, evident from the increased mRNA expression of cytokines such as *IL-1β* and *IL-8*, whereas the *Lactobacillus crispatus* supplementation reduced the *IL-1β* expression, proving more effective than its heat-killed counterpart. Tight-junction components such as *OLDN*, *CLDN*, and *ZO-1*, which are crucial for epithelial barrier integrity, are downregulated in oviductal inflammation, affecting the egg production and oviduct health through weakened mucosal barriers [30,31,32]. However, *Lactobacillus acidophilus* and *B. subtilis* have been shown to improve mucosal barriers via tight junction protein upregulation. *Lactobacillus crispatus* supplementation was found to enhance the *CLDN* and *ZO-1* expression, suggesting that improved mucosal barriers may be linked to reduced pro-inflammatory cytokine expression. Biochemical and metabolic indicators in the serum and uterus directly reflect the metabolism and health status in laying hens [33]. In this study, both fresh and heat-killed *Lactobacillus crispatus* significantly reduced the levels of inflammatory factors such as IL-1β, IL-6, IL-8, and TNF-α, indicating their potential to regulate the immune functions in laying hens. Concurrently, a recent study in mice demonstrated that the immune responses induced by different heat-killed Lactobacillus species varied, with *L. paracasei* exhibiting the highest IL-12 secretion induction capacity, outperforming species such as *L. reuteri*, *L. casei*, and *L. plantarum* [34]. Consequently, supplementation with either fresh or heat-killed *Lactobacillus crispatus* alleviates oviductal inflammation in laying hens. This therapeutic effect may be attributed to the retained capacity of heat-killed *lactobacilli* to produce secretory IgA and other anti-inflammatory metabolites, which have been shown to significantly reduce inflammation and inhibit the colonization of pathogens such as *S. thermophilus* and *E. coli* [35,36]. These findings are consistent with prior research on mice, which confirmed that *Lactobacillus crispatus* could prevent uterine apoptosis and inflammation [37]. Further supporting these results, Guo et al.’s research on the effects of fermented feed suggests that *Lactobacillus crispatus* supplementation can alleviate reproductive inflammation and strengthen mucosal barriers [13].

In this study, we found that the *Lactobacillus crispatus* supplementation reduced the community richness, likely through the proliferation of cecal probiotics such as *Faecalibacterium* and *Megamonas*, which can inhibit harmful microbes. Consistent with prior research, the laying hen cecal microbiome was predominantly composed of *Firmicutes*, *Bacteroidetes*, and *Actinobacteria* [25,33,38,39]. Short-chain fatty acids (SCFAs), which are crucial bacterial metabolites, including acetate, butyrate, and propionate, serve as direct energy sources for the host and play key roles in modulating inflammation, with butyrate notably suppressing pro-inflammatory effectors in macrophages [40,41,42]. *Ruminococcus* within *Firmicutes* can produce short-chain fatty acids, such as acetate and butyrate [43]. *Ruminococcus torques*, part of the Clostridia class, metabolize indigestible polysaccharides and amino acids to produce beneficial compounds, such as secondary bile acids, and are linked to a lower risk of female infertility [44,45]. The acetate and butyrate produced by *Megamonas* act as anti-inflammatory compounds and are thus positively associated with intestinal health [46]. Several studies have suggested that *Faecalibacteriumc* contributes to immune homeostasis by producing butyrate and the anti-inflammatory cytokine IL-10 [47,48]. Indeed, in our study, there was an increasing IL-10 expression tendency observed in the uterus; however, further study on its expression in the cecum is needed. The primary phyla observed in the uterus and isthmus were *Firmicutes*, *Actinobacteriota*, *Proteobacteria*, and *Bacteroidetes*, with changes in their relative abundances following the *Lactobacillus crispatus* supplementation [4,49,50]. Notably, *Lactobacillus crispatus* led to a higher *Firmicutes*–*Bacteroidetes* ratio in the uterus microbiome, associated with increased food energy absorption [51,52]. Additionally, *Actinobacteriota*, especially *Rhodococcus*, saw a significant increase in the uterus and isthmus, aligning with findings for the aged uteri of laying hens [53]. *Actinobacteria*, known for the anti-inflammatory, immunomodulatory, and antibacterial properties of its metabolites, plays a crucial role in maintaining host immune homeostasis [54,55].

The LEfSe analysis revealed that the relative abundances of *Rhodococcus* within *Actinobacteriota*, *Ochrobactrum* within *Proteobacteria*, and *Delftia* within *Pseudomonadota* were significantly enriched in the *Lactobacillus crispatus* supplementation group in the uterus. This was likely because adding *Lactobacillus crispatus* significantly improved the hosts’ nutritional metabolism and intestinal health, thereby regulating their immune functions. Through the gut–reproductive tract axis, it also affected the health of the reproductive tract. *Ochrobactrum* and *Rhodococcus* can degrade lignocellulose biomass into simple sugars [56]. Ochrobactrum is also linked to an improved gut immune function, evidenced by its correlation with IL-10 in the cecum, explaining the cecal morphology improvement post-supplementation [39]. As a probiotic, *Rhodococcus SM2* protects aquatic animals from infectious diseases, as its trehalolipids have immunoregulatory activities [57,58]. Studies have confirmed the diverse immunoregulatory activities of trehalolipids produced by nonpathogenic *Rhodococcus* [59]. *Rhodococcus* strains degrade toxic chemicals, including phenolic compounds, while Delftia efficiently degrades aromatic compounds such as naphenol [60,61], suggesting that *Lactobacillus crispatus* supplementation may mitigate oviductal inflammation effects through the degradation activities of *Rhodococcus* and *Delftia*. The higher laying rates in the LAC and HLAC groups may be attributed to other beneficial bacteria, such as Romboutsia, known for its antioxidant and anti-inflammatory effects in mice, and Bacillus, which modulates gut microbiota and increases SCFA levels, enhancing the immunity and intestinal homeostasis in poultry [62,63,64]. The enrichment of *Clostridium_sensu_stricto_1*, a major butyrate producer, contrasted with that of *Achromobacter*, associated with lung structure damage and chronic airway infections, in the OI group. The presence of pathogenic bacteria such as *Helicobacter*, *Collinsella*, and *Streptococcus* in the OI group underscores *Lactobacillus crispatus*’s potential for improving chicken health by reducing harmful genera [65,66,67,68,69,70]. In addition to the *Rhodococcus*, *Ochrobactrum*, and *Delftia* enrichment, the *Lactobacillus crispatus* supplementation also enriched *OLB8* and *Ralstonia*, with the *Bacteroidetes* bacterium *OLB8* enhancing the carbon and energy metabolism through polysaccharide and protein degradation, further supporting the supplement’s beneficial effects on poultry health [71,72].

## 5. Conclusions

We discovered that supplementing laying hens with either fresh or heat-killed *Lactobacillus crispatus* similarly reduced inflammation, significantly improving the oviductal inflammation. Although the live *Lactobacillus crispatus* demonstrated a slightly more pronounced effect on the oviduct health and microbiota environment, both forms were effective. Overall, this research provides valuable insights into mitigating oviductal inflammation in laying hens.

## Figures and Tables

**Table 1:** Ingredient composition and nutrient content of basal diet (%, DM).

Ingredients	Percentage (%)
Corn	65.05
Soybean meal	24.20
Limestone	8.20
Dicalcium phosphate	1.70
Salt	0.30
DL-methionine	0.12
Choline chloride	0.10
Mineral premix ^1^	0.30
Vitamin premix ^2^	0.03
Total	100
Nutrient levels ^3^	
Metabolizable energy (Mcal/kg)	2.69
Crude protein	16.04
Calcium	3.60
Non-Phosphorus	0.39
Methionine	0.38
Lysine	0.78
Threonine	0.59
Tryptophan	0.16

^1^ Mineral premix provided (per kg of diet): Cu (CuSO_4_·5H_2_O), 6.8 mg; Fe (FeSO_4_·7H_2_O), 66 mg; Zn (ZnSO_4_·7H_2_O), 83 mg; Mn (MnSO_4_·H_2_O), 80 mg; I (KI), 1 mg; ^2^ Vitamin premix supplied (per kg of diet): vitamin A, 117,000 IU; vitamin D3, 3600 IU; vitamin E, 21 IU; vitamin K3, 4.2 mg; vitamin B1, 3 mg; vitamin B2, 10.2 mg; folic acid, 0.9 mg; calcium pantothenate, 15 mg; niacin, 45 mg; vitamin B6, 5.4 mg; vitamin B12, 24 μg; biotin, 150 μg; ^3^ Contents of Calcium and Phosphorus were analyzed.

## Data Availability

The raw data supporting the conclusions of this article will be made available by the authors on request.

## Supplementary Materials

The following supporting information can be downloaded at: https://www.mdpi.com/article/10.3390/microorganisms12081559/s1, Table S1: Sequence of primers pairs used in real-time quantitative PCR.
