# Supplementary material for: Lactobacillus crispatus-Mediated Gut–Reproductive Tract Axis-Alleviated Microbial Dysbiosis and Oviductal Inflammation in a Laying Hen Model
Source: Microorganisms. 2024 Jul 30;12(8):1559. doi: 10.3390/microorganisms12081559 (PMC11356123; doi:10.3390/microorganisms12081559)
Supplement: Supplementary file 1 [file microorganisms-12-01559-s001.zip › microorganisms-2984386-supplementary.pdf]

Table S1. Sequence of primers pairs used in real-time quantitative PCR.

Gene Name	Primer Sequence (5′–3′)
cIL-1β	F: ACTGGGCATCAAGGGCTA<br>R: GGTAGAAGATGAAGCGGGTC
cIL-8	F: ATGAACGGCAAGCTTGGAGCTG<br>R: TCCAAGCACCTCTCTTCCATCC
cIL-10	F: GCTGCCAAGCCCTGTT<br>R: CCTCAAACTTCACCCTCA
cZO-1	F: CTTCAGGTGTTTCTCTTCCTCCTC<br>R: CTGTGGTTTCATGGCTGGATC
cOLDN	F: ACGGCAGCACCTACCTCAA<br>R: GGGCGAAGAAGCAGATGAG
cCLDN	F: CATACTCCTGGGTCTGGTTGGT<br>R: GACAGCCATCCGCATCTTCT
cGAPDH	F: GTCCAAGTGGTGGCCATCAA<br>R: GCTGAGGGAGCTGAGATGAT
